# Experiences of facilitators regarding the extended curriculum programme offered at a higher education institution in the province of KwaZulu-Natal in South Africa

**DOI:** 10.4102/curationis.v41i1.1895

**Published:** 2018-09-20

**Authors:** Maureen N. Sibiya, Hazel T. Mahlanze

**Affiliations:** 1 Faculty of Health Sciences, Durban University of Technology, South Africa; 2 Department of Nursing, Durban University of Technology, South Africa

## Abstract

**Background:**

Much like the rest of the world, student access and success are primary concerns of the South African higher education institutions, especially in the face of data that suggest that up to 50% of students do not successfully complete their course of study. Despite compulsory and free basic education for all South Africans, and increased government funding for education, there has been little impact on learner performance and the majority of primary schools remain poor. To improve access and success and in keeping with international practice, the Department of Nursing at the selected university of technology in 2013 offered for the first time the extended curriculum programme (ECP). To date, the impact of the programme has never been evaluated.

**Objectives:**

The aim of the study was to explore the experiences of the facilitators regarding ECP in the undergraduate nursing programme.

**Method:**

Guided by this, the current article describes a qualitative exploration of the experiences of six purposively selected facilitators regarding ECP in the Department of Nursing. In-depth interviews were conducted with the ECP facilitators. Tesch’s method was used to analyse the data.

**Results:**

Four main themes emerged from the data: stigmatisation and lack of confidence, lack of self-will, additional workload of facilitators and gradual improvement of students’ performance. The participants reported that although students displayed and verbalised negative attitude towards the ECP, the performance of students showed gradual improvement and thus a need to continue to offer the programme to increase access and success in higher education institutions.

**Conclusion:**

It was concluded that ECP should continue to increase access and success in higher education institutions; however, there is a need for additional resources to support ECP students.

## Background of the study

Student access and success are primary concerns of higher education institutions (HEIs) throughout the world (Lewin & Mawoyo 2014:3). This is unsurprising given the challenges that continue to be evident across global institutions. Some literature (Department of Higher Education and Training [DHET] 2016:14; Lewin & Mawoyo 2014:12; National Planning Commission 2011:271; Wangenge-Ouma 2013:9; Weybright et al. 2017:1) including the Council on Higher Education (CHE 2013:47) presents evidence that shows dropout and non-completion rates to be as high as 50% across many universities in South Africa. Success rates seem to differ from one context to another, with some suggesting that material advantages are a central factor. In South Africa, access and success are profoundly linked to the social and political context within which universities operate. South Africa has high attrition rates not only in HEIs but also in basic education (Grossen, Globler & Lacante 2017:1; Lewin & Mawoyo 2014:23). Approximately 60% of the learners who enter the schooling system complete Grade 12, while 40% of the learners drop out of the system after repeated failure (Grossen et al. 2017:7; Weybright et al. 2017:1). There are about 50% of both undergraduate and postgraduate students who drop out before completing their studies (Wangenge-Ouma 2013:9). The literature shows that South Africa’s average graduation rate is 15% (Lewin & Mawoyo 2014:12), and completion rates between white and black students are significantly different; black completion rate is less than half of the white completion rate (CHE 2013:59).

The socio-demographic profile of students plays a major role in academic performance both at school and in higher education. Some researchers argue that socio-economic factors are the most reliable indicators of higher education achievement (Bass 2011:49; CHE 2013:59; Dhunpath & Vithal 2014:41; Ross 2010: 460). The White Paper for Post-School Education and Training noted that, despite the advances made since the advent of democracy, the education system continues to replicate the divisions of the past. The institutional landscape is still reminiscent of apartheid, with disadvantaged institutions still disadvantaged in terms of infrastructure, teaching facilities and staffing (DHET 2013:1). Basic education is a right for all citizens (Republic of South Africa 1996:14). Despite compulsory and free education for all South Africans, and increased government funding for education, there has been little impact on learner performance; therefore, the majority of primary schools remain poor (Weybright et al. 2017:8). Although historically black and previously disadvantaged schools make up 80% of the country’s secondary schools, these schools produce only 20% of students who qualify for university (Wangenge-Ouma 2013:9). With the widening of access to South African HEIs, a change in the demographics of students is noted. Young people from various backgrounds are now able to access HEIs (CHE 2010:3; Lewin & Mawoyo 2014:23). According to the National Development Plan, the South African post-school system is not well designed to meet the skills development needs of either the youth or the economy (National Planning Commission 2011:316); hence, a need for a programme that supports underprepared students to improve access and success in HEIs.

Extended curriculum programme (ECP) is a South African higher education intervention with much significance for the case for the structural curriculum reform (CHE 2013:70). The purpose of the ECP is to create the curriculum space needed to enable talented but underprepared students to achieve sound foundations of success in higher education. A report that was commissioned by the World Bank states that this method of students’ support is widely used across the globe to address the articulation gap to provide access that is meaningful (Fisher & Scott 2011:25).

According to Lewin and Mawoyo (2014:3), student access and success must be understood in historical terms. Until the early 1990s, black students entered higher education in low numbers. In the early 1990s, a massive expansion of black student enrolment in higher education occurred (Badat 2009:466; Bunting 2002:40). Black student numbers increased from 55% in 1994 to 81% in 2011 (Lewin & Mawoyo 2014:23). While the South African higher education system has arguably performed well in terms of opening up access, the same cannot be said about retention.

Council on Higher Education (2010:49) requires that programmes promote graduates’ successful integration into the world of work and enable them to make meaningful contributions in contexts of development. It is therefore imperative that innovative curricular, teaching, learning and assessment practices are developed to prepare graduates to meet these global trends. In 2013, the Undergraduate (UG) Nursing Programme within the Department of Nursing at the selected university of technology offered for the first time the ECP. The UG Nursing Programme is relatively small, with 10 full-time lecturers including the programme coordinator, three part-time lecturers and nine clinical instructors. A group of not less than 100 mainstream students and 20 ECP students are admitted annually. At the time of this study, 39 students were registered in the first and second year of the ECP. Three students deregistered in 2013 and one in 2014.

Standardised Assessment Tests for Access and Placement (SATAP) are used for selection and placement of all applicants. The focus is on cognitive process and skills that underpin tasks more than on discipline content. Tests, which are primarily multiple choice questions (MCQs), are designed to assess competencies in reading and writing modes only. Applicants who obtain scores below 50% are placed in the ECP. The programme is offered over 5 years to enhance student development and to improve the students’ chances of successful completion. During the first 3 years, the students register for a few core nursing modules and essentials of professional practice (EPP). Essentially the professional practice module consists of academic literacy subjects, for example computer literacy, English literacy and information literacy. The EPP subjects are designed to develop and support the students’ capability to cope with the core subjects. Exploration of the facilitators’ experiences regarding ECP will determine if the ECP should continue to increase access and success in HEIs. Exploring the experiences of the facilitators will shed light in determining the strengths and the weaknesses of the ECP thus far. It is hoped that the challenges faced by facilitators and students will be elicited; faculty and executive management commitment to the programme will be evaluated as well as curriculum and academic development issues pertaining to the ECP will be highlighted and recommendations made to improve success of the ECP.

## Aim

Therefore, the aim of the study was to explore the experiences of the facilitators regarding ECP in the UG Nursing Programme.

## Research methodology

### Design

A single case study, using a qualitative research design, was used to guide the study. Case studies are used by researchers to thoroughly explore a programme, an event, an activity, a process or one or more individuals, but families, groups, institutions and other social units may also be the focus (Yin 2009). A case study design was chosen for this study because it will allow the researchers to explore the experiences of the ECP facilitators in one programme within the context of the research. Case studies have shown to be very useful in health and social sciences and are used when exploring the how and why of a situation when variables cannot be controlled (Yin 2009:17).

### Study location

The UG Nursing Programme is located in a semi-urban township of Imbali in the province of KwaZulu-Natal (KZN). Most of the students are from peri-urban areas, rural areas or previously disadvantaged groups. Students are largely black South Africans and from various provinces. The UG Nursing Programme offers the 4-year bachelor’s degree as well as the ECP, which is a bachelor’s degree that extends over a period of 5 years to provide a curriculum response to improve student access and success (Lewin & Mawoyo 2014:72).

### Sample process

The participants were purposively selected. Polit and Beck (2012:279) describe purposive sampling as a method often used when the researcher wants a sample of experts. This method of sampling was chosen to enable the researcher to mix the ECP facilitators and to generate appropriate, information-rich and useful data from the participants. The number of participants in a qualitative study is adequate only when saturation of information is achieved in the study area because fresh data no longer spark new insights or reveal new properties (Creswell 2014:297). The sample comprised six consenting ECP facilitators. Five of the facilitators have been involved with the ECP since its inception in 2013, and one for 2 years.

### Data collection and analysis

In-depth interviews were conducted with the ECP facilitators. The key research question that was asked during the interviews was: ‘As the facilitator in the programme, what are your experiences regarding ECP in the UG Nursing Programme’. Participants’ permission was obtained to audio-tape interviews. Notes were written during the interview. Each interview session lasted 30–45 min. Interviews were conducted by the researcher on a day that suited the participants. An interview guide was used to facilitate the discussions. Tesch’s method was used to analyse the data. Tesch’s eight-step procedure of data analysis was applied to analyse data (Tesch 1990 in Creswell 2009:186). The researcher spent one week listening to all the voice recordings to familiarise herself with the data recorded before data transcription began. The researcher coded every script in order to develop a comprehensive framework for analysis. The comprehensive framework was then used for more detailed coding and thematic content analysis. Emerging themes and sub-themes were thereafter identified from the raw data.

Data collection took place in March–April 2015. An information session was held with the participants, and thereafter written informed consent was obtained. Participants were informed that participation in the study was voluntary. Interviews were conducted at the facilitators’ offices to minimise any interference during the session.

### Trustworthiness

Criteria as set out by Lincoln and Guba (1985) in Polit and Beck (2012:584) were used to establish the trustworthiness of qualitative data. To ensure credibility in this study, notes were written during the interview, and detailed notes were written immediately after the interview. An audit trail was maintained through safekeeping of raw data of each interview for future reference. Following the transcription of the voice-recorded interviews, each participant was given an opportunity to review the transcribed interview and was asked to confirm if the notes are of true reflection of his or her views regarding the supervision experiences. Voice recordings were done to reflect the participant’s voice. To ensure transferability, there was rich and thorough description of the research setting and of the research processes.

### Ethical considerations

Prior to the commencement of data collection, ethical clearance was obtained from the university ethics committee (REC 5/15). Permission to conduct the study was obtained from the Research Director at Durban University of Technology and Deputy Head of the Department of Nursing. An information letter was provided to the participants and written informed consent was obtained from all participants. The participants were assigned a code to maintain confidentiality. All data will be stored securely by the researcher for 5 years.

## Results

### Demographic information

A total of six ECP facilitators participated in the study. Of the six facilitators, three facilitated core nursing modules and three facilitated the EPP modules. The study consisted of five female participants and one male. Participants had more than 5 years’ experience in higher education.

The following four major themes emerged from the study findings:

- stigmatisation and lack of confidence
- lack of self-will
- additional workload of facilitators
- gradual improvement of students’ performance.

Table 1 provides the major themes and sub-themes that emerged from the findings.

**TABLE 1:** Themes and sub-themes.

Theme	Sub-theme
Stigmatisation and lack of confidence	Feeling unhappy
	Lack of confidence
	Lack of acceptance
Lack of self-will	Needs pushing
	Cannot work independently
	Lack of critical thinking skills
Additional workload of facilitators	Unnecessary strain to the facilitators
	Demanded from students to be spoon-fed
Gradual improvement of students’ performance	Gradually improved performance
	Improvement in assertiveness
	Support by tutors

### Stigmatisation and lack of confidence

The majority of the participants reported that the students had negative attitude towards the ECP. The participants further reported that students felt stigmatised and lacked confidence because of placement in the ECP. This is noted in the quotations below:

‘Students verbalized that they are not happy that they are in the ECP and they report that this affect them psychologically.’ (Participant 1, male, 6 years’ experience)

‘The attitude of some of the students was mainly the hindrance in their academic writing progress. The majority of the students felt that they should have never been part of the ECP and as a result, they were not fully engaged in classroom activities and most students had negative attitude towards learning.’ (Participant 4, female, 8 years’ experience)

‘Most students that were recommended to attend the one-to-one consultation at the Writing Centre did not attend.’ (Participant 3, female, 7 years’ experience)

‘They felt isolated and discriminated irrespective of explanations given to them.’ (Participant 6, female, 6 years’ experience)

‘Students felt sorry for themselves. They displayed lack of confidence.’ (Participant 1, male, 6 years’ experience)

‘They felt stigmatized by other students and were judged as not clever.’ (Participant 2, female, 12 years’ experience)

### Lack of self-will

The facilitators were of the opinion that the majority of the ECP students lacked self-will. They reported that students lacked critical thinking skills as shown in the excerpts below:

‘…they need constant pushing. They do not want to work independently.’ (Participant 5, female, 6 years’ experience)

‘The majority of the students displayed slow reception of instructions. One has to repeat the instruction more than once before they get it.’ (Participant 4, female, 8 years’ experience)

‘Based on their performance, one can conclude that the ECP students have poor critical thinking skills. They have inability to articulate knowledge.’ (Participant 1, male, 6 years’ experience)

### Additional workload of facilitators

Because of students’ negative attitude towards ECP and limited critical thinking skills, the participants raised some concerns that the students placed additional workload on them as noted in the quotes below:

‘They demand to be spoon-fed and sometimes want more help from lecturers and it becomes too much for the lecturers.’ (Participant 2, female, 12years’ experience)

‘Their negative attitude towards the programme puts unnecessary strain to the facilitators since they have to put double the effort in changing their mindset.’ (Participant 6, female, 6 years’ experience)

### Gradual improvement of students’ performance

All the facilitators reported that they had confidence that the ECP would assist the students to improve learning. This is noted in the excerpts below:

‘This year there is improvement in their assertiveness and readiness to learn unlike the past.’ (Participant 4, female, 8 years’ experience)

‘In their third year of study, I see transformation and an attitude favourable for learning.’ (Participant 3, female, 7 years’ experience)

‘The appointed tutors will hopefully help with motivation and support.’ (Participant 1, male, 6 years’ experience)

‘Grasping of blackboard was difficult in their first year, but 2^nd^ year enjoyed it and they work independently.’ (Participant 5, female, 6 years’ experience)

## Discussion

In this study, participants reported that students felt stigmatised and lacked confidence because of placement in the ECP. It is worth noting that the CHE concurs that the problems inherent in the current ECP model, arising primarily from its minority and marginalised status, impose intractable limitations on its success. This is contrary to the purpose of the ECP as previously stated that it is to support the underprepared students to achieve sound foundations of success in higher education. The CHE (2013:70) argues that the structural curriculum reform that takes account of students’ educational backgrounds can positively influence student performance. To address this challenge, the DHET initiated the Academic Development Programmes that are aimed to create access opportunities and support systems for talented but disadvantaged South African students (CHE 2013:70).

The findings of the current study are supported by the CHE that argues that because the ECP students are significantly more at risk than mainstream entrants, lower performance levels can be expected, even with the additional support offered (CHE 2013:82). The foundation students’ success rates are comparable with those of the whole student body, particularly in view of their different risk profiles. This indicates that if the articulation gap is addressed, students who are underprepared for existing mainstream curricula are able to perform comparably with their better prepared peers (CHE 2013:78). In his study on teachings from consumer behaviour applicable to higher education, Du Preez (2015:152) recommends an investment in student support structures that cater for academic and socio-psychological needs, as more students are underprepared for higher education. The growing number of first-generation students deserves special attention as very little is known about the unique academic and socio-psychological challenges that they are facing upon entry into higher education.

Participants raised some concerns that the ECP students placed additional workload on them. The CHE (2013:57) argues that learning depends on whether students can and do respond positively to the educational process in HEIs. On the other side, educational expertise, at different levels and in different forms, is necessary for successfully addressing central challenges in South African higher education, for substantially improving the current performance patterns and thus for meeting the country’s high-level human resource needs. Given the nature of the HEIs in South Africa that have a challenge of low success rates, increasing positive engagement with educational capacity building depends on strengthening the recognition and status of teaching expertise. The findings of the study that was conducted by Hans (2014:100) revealed that lack of skills and expertise was one of the shortcomings of the support unit of the ECP. The staff could not render all services expected from an education support unit, including identifying the intrinsic and extrinsic barriers to learning in the educational environment. Capacity building calls for the provision of effective professional development opportunities in the sector. Faculties who run extended programmes must ensure integration and communication between ECP facilitators and the mainstream staff members so that the latter have a sense of belonging as valuable contributors (Garraway 2009:49). Staff involved in the ECP must undergo some training in the nature and purpose of the extended programme and in how to promote student engagement and academic support (Garraway 2009:53). Extended curriculum programme funding is based on a 3-year cycle and this makes the stability of staff difficult and may affect the delivery of modules; making funding permanent will afford ECP programmes permanent staff who will, in turn, be motivated to stay and improve the programme (Garraway 2009:49).

All participants shared the same sentiments that the ECP will result in gradual improvement of students’ performance. These findings corroborate the results of the quantitative data on ECP that were gathered by the DHET from institutional annual reports 2007–2011, which revealed that the foundation students’ success rates are comparable with those of the whole student body, particularly in view of their different risk profiles. This indicates that if the articulation gap is addressed, students who are underprepared for existing mainstream curricula are able to perform comparably with their better prepared peers (CHE 2013:78). Du Preez (2015:147) argues that students who are currently in higher education have inflated levels of self-confidence. He further states that they enjoy a high-energy learning environment, have high optimum stimulation levels, seek developmental opportunities and enjoy being challenged. This argument supports the findings of this study which highlight the need for the ECP.

## Limitations

This study was conducted in one selected HEI in KZN; thus, it is unlikely that the sample represents other HEIs. The study was qualitative and thus the results cannot be generalised; there is a need to conduct similar studies in other HEIs.

## Implication of findings on wider practice and future research possibilities

An evaluation study of the ECP is necessary that will explore throughput rates of students who have been through the programme in order to investigate and understand the successes and challenges of the ECP. This research was conducted in one academic programme; future research should ideally include a representative sample of other programmes who offer the ECP at the HEI under study.

## Recommendations

The participants recommended that there should be appointment of additional tutors to support ECP students. They also recommended an intense orientation programme at the beginning of the year for the ECP students to enhance awareness of the benefits of the programme.

## Conclusion

The findings of the study, which was aimed at exploring the experiences of the ECP facilitators, revealed that the participants reported that although students displayed and verbalised negative attitude towards the ECP, their performance showed gradual improvement and thus a need to continue to offer the programme to increase access and success in HEIs.
